# A Proof-of-Concept Solution for Co-locating 2D Histology Images in 3D for Histology-to-CT and MR Image Registration: Closing the Loop for Bone Sarcoma Treatment Planning

**DOI:** 10.1007/s10278-025-01426-5

**Published:** 2025-02-26

**Authors:** Robert Phillips, Constantine Zakkaroff, Keren Dittmer, Nicholas Robilliard, Kenzie Baer, Anthony Butler

**Affiliations:** 1 The University of Otago - Canterbury, Christchurch, New Zealand; 2 University of Canterbury, Christchurch, New Zealand; 3 Massey University, Palmerston North, New Zealand; 4 Te Whatu Ora (Canterbury Health Labs), Christchurch, New Zealand; 5 Te Whatu Ora, Christchurch, New Zealand

**Keywords:** Radiopathology, Patient-specific treatment, Histopathology digitisation, Orthopaedic oncology, Translational research

## Abstract

This work presents a proof-of-concept solution designed to facilitate more accurate radiographic feature characterisation in pre-surgical CT/MR volumes. The solution involves 3D co-location of 2D digital histology slides within *ex-vivo*, tumour tissue CT volumes. Initially, laboratory dissection measurements seed the placement of histology slices in corresponding CT volumes, followed by in-plane point-based registration of bone in histology images to the bone in CT. Validation using six bisected canine humerus *ex-vivo* CT datasets indicated a plane misalignment of 0.19 ± 1.8 mm. User input sensitivity was assessed at 0.08 ± 0.2 mm for plane translation and 0–1.6° deviation. These results show a similar magnitude of error to related prostate histology co-location work. Although demonstrated with a femoral canine sarcoma tumour, this solution can be generalised to various orthopaedic geometries and sites. It supports high-fidelity histology image co-location to improve understanding of tissue characterisation accuracy in clinical radiology. This solution requires only minimal adjustment to routine workflows. By integrating histology insights earlier in the presentation-diagnosis-planning-surgery-recovery loop, this solution guides data co-location to support the continued evaluation of safe pre-surgical margins.

## Introduction

We present a computational and tissue processing solution for the co-location of 2D histology images and 3D sarcoma radiology volumes. The solution uses two CT scans of each half of a bisected tumour specimen following surgery and incorporates offset measurements in laboratory processing. We outline a sequence of operations for further registration with pre-surgical CT and MR. The combination of radiographic datasets with co-located histology images and label maps will support image interpretation and segmentation confidence when making subsequent surgical resection margin decisions.

Histology slides, radiology, and an improved laboratory workflow from previous experimental work on bone sarcoma-affected limbs [1] were used to develop and refine computations. Data from an additional canine bone sarcoma was collected after routine veterinary surgery to demonstrate the continuity of the co-location solution.

**Fig. 1:**
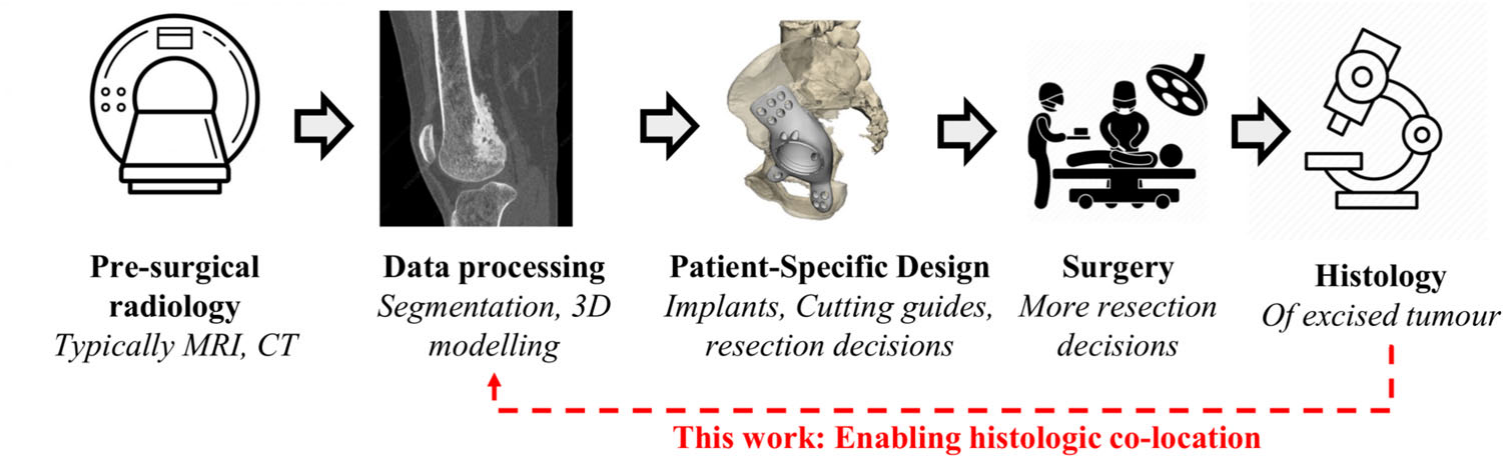
Reference human patient-specific clinical workflow for bone sarcoma

### Challenges and Considerations in the Treatment of Bone Sarcomas

Bone sarcomas are some of the most challenging tumours to treat [2, 3]. Treatment and management of these tumours are less well understood because they are rare and heterogeneous, consisting of a variety of cell types, sub-populations, and tissue substructures [4]. They are over-represented in younger populations and, if not fatal, often impact mobility and quality of life [3, 5]. Orthopaedic surgery is indicated for all curative treatments in conjunction with chemoradiation in most cases [6, 7]. Complex procedures, such as hemipelvectomy, may be necessary in the pelvis or other complex anatomy [8]. Despite the inclusion of indicated multi-agent systemic chemotherapy, overall five-year survival rates for the most common bone sarcoma in the pelvis have not improved much beyond 27% [3, 9].

Resection margins are applied to ensure full removal of the tumour. However, the loss of healthy tissue with these margins affects patient outcomes. The recommended curative treatment is a *wide* resection, which aims to include 10 mm or 20 mm of healthy tissue around the removed tumour [6, 10]. Sometimes wider margins are taken due to factors such as the patient’s risk profile, sarcoma sub-type or geometry of the surgical cuts. Unfortunately, damage to muscles, nerves and blood vessels resulting from healthy tissue removal impairs post-surgical mobility and body function. These complications can burden the patient, family, the community, and the health system [11].

Clinical teams face difficult decisions regarding resection margins. They must remove healthy functional tissue to ensure surgical success while also attempting to minimise harm from loss of the same healthy tissue [12–15]. Unfortunately, there is limited data to predict the surgical success of different resection options. This forces clinical teams to make important decisions with minimal quantitative guidance.

Patient-specific technology shows promise for reducing surgical margins. For example, computer-aided design (CAD) models help sarcoma treatment teams *salvage* healthy tissue, especially in areas with complex anatomy [16]. However, prospective studies face challenges in evaluating the efficacy of custom implants, patient-specific instruments (PSI) and cutting guides, or surgical navigation technology with traditional orthopaedic benchmarks due to limited sample availability [2, 17, 18].

The need for more samples presents a challenge. Traditional orthopaedic oncology research often spans decades before confidently recommending new surgical techniques [6, 19, 20]. However, the rapid evolution of salvage technology, since the advent of 3D printers and better access to computers, highlights the need for a solution to understand the inherent risk of different surgical techniques. High-fidelity data has been proposed and could be used to assess sarcoma treatment [21]. Unfortunately, the utilisation of such data is limited by constraints in the current post-surgical analysis reporting [22, 23].

Medical image segmentation and 3D modelling Fig. 1 are common techniques used in almost all patient-specific technologies [24]. Using segmented CAD models to guide resection decisions is immensely valuable for clinical teams. However, this process places significant reliance on the engineers and machines performing the segmentation and on the radiologists and surgeons who check the output CAD models. The increasing reliance on CAD models to determine resection margins introduces a limitation, as tumour segmentation remains subjective and difficult to quantify.

Some studies have evaluated the radiological presentation of sarcoma in one dimension using the clinical standard of histopathology, the diagnosis of the disease by microscopic evaluation [25–28]. Although these radiographic evaluation techniques are clinically relevant, they only capture a fraction of the information contained in the histology. Furthermore, no histology registration studies have been found suitable for pelvic tissue or other complex, curved anatomy. Areas where resection margin evaluation could greatly benefit patients.

Co-location of 2D histology images in 3D radiology volumes has been shown to be effective for validating tissue boundaries and tumour margins for soft tissue [29]. Many novel techniques have been developed to co-register *ex-vivo* histology and *in-vivo* MR [30–32]. However, few published techniques are suitable for bone, with the most notable requiring cryogenically frozen mice [33]. Cryogenesis is not always practical for larger clinical sarcoma.

In contrast, this paper outlines a solution to co-locate histological images and register them to medical image volumes for bone sarcoma. The proposed solution accommodates variations in shape, size, and tissue heterogeneity and can be performed with minimal disruption to most clinical pathology workflows.

#### Accuracy of Radiologic Segmentation

Clinical radiology is widely used in innovative salvage techniques [23]. CAD models are increasingly used to determine resection margins before surgery. These margins are often integrated into printed devices, tools, or digital surgical navigation systems [16, 34]. However, a *critical assessment* of resection margins and therapy techniques is needed to realise the full opportunities of advanced surgical technology [23], of which segmentation is an integral part.

The segmentation of sarcoma can vary due to radiology image resolution, artefacts like partial volume averaging, inhomogeneity, motion, or different user interpretations. Although medical image resolution and radiology protocols for diagnosis and segmentation are continually evolving [35], their interpretation remains fundamentally subjective. Technology providers are developing artefact reduction technologies for CT and MR, although these come with their own challenges. Other recent research focuses on automating model segmentation to reduce human variation and support clinical translation [36]. However, all of these initiatives face the same issue: it is difficult to quantify how well individual image features approximate the boundaries of physical tissue [21].

Co-location of 2D histology and 3D radiology image features is valuable for quantifying image segmentation and radiomics as it links microscopic tissue properties to macroscopic information [29]. Our solution supports histology image co-location within radiology volumes, facilitating quantitative and semiquantitative image processing for bone sarcoma.

### Histology Registration Current State-of-the-Art

Histopathology is the clinical standard for tumour diagnosis and is used to guide post-surgical treatment [37, 38]. It uses histology techniques to assess tumour boundaries. Despite its relatively low cost, histology provides a substantial amount of high-fidelity data and is expected to remain a cornerstone of pathology [38, 39].

Histology techniques involve dissecting resected specimens, fixing them to prevent autolysis, and performing several immersion steps. Thin shavings of each dissection are then stained on glass slides and analysed under a microscope.

The histological literature describes how slides should quantitatively be measured and interpreted [40]. Several classification systems describe what healthy tissue margins mean for post-surgical tumour treatment. The Residual (or *R*) classification in the Tumour, Node, Metastasis (TNM) system is a common metric used to check surgical success [17, 22].

Increasing digitisation provides very high-fidelity images for use in treatment but also processing beyond clinical workflows [37, 39]. During the last three decades, successful co-location of microscopic histology and registration with macroscopic radiology volumes has been performed mainly with soft tissues [29]. Histology can produce a discontinuous stack of images with an effective pixel size of 5 $\mu m$ by 5 $\mu m$ for 4 $\mu m$ thick slices of tissue, creating a volume with tiny voxels if shaved in parallel [41]. In contrast, MR has a typical voxel size of 0.4 $\times$ 0.4 $\times$ 4.0 $mm^3$ [41].

Unfortunately, spatial co-location of human bone sarcoma histology with MR remains unattainable with current solutions.

This paper employs a novel pathology processing approach to assist the co-location of histological images within bone sarcoma radiology. It can stand alone, or be added to the start of existing open-source histology image co-location solutions [41].

### Anticipating Human Clinical Use Early

Registration between *in-vivo* imaging modalities, such as CT and MR, is well established. And augmentation with histology can make high-fidelity information available at a low cost [29]. However, it is challenging to accurately co-locate 2D histology images within a 3D volume in established clinical environments [38]. Previous research has highlighted three key requirements of any histology co-location solution: (1) to permit specimen slicing and dissecting (‘cut-up’) in line with pathology protocols; (2) to provide co-location accuracy that is robust to the variation of tissue appearance on radiology and histology; and (3) to provide a quantitative evaluation of co-location error [31].

The collection of high-quality 3D bone histology datasets should prioritise the simple adaptation of existing pathology protocols before overhauling entire systems or requiring specialised equipment. It is noted that (a) pathology protocols vary due to numerous factors, such as equipment availability and staffing levels. (b) Bone sarcoma is highly variable in shape and size. (c) Bone sarcoma is rare, making it crucial that the solution is usable in as many sarcoma centres as possible. Minimising protocol change improves resilience by leveraging the evolved knowledge and expertise within institutions.

While previous studies have explored histology co-location in the context of the prostate, this work focuses on bone anatomy and integration with the reference sarcoma workflow Fig. 1. Large bone specimens are slow to fix and require prior dissection to ensure proper penetration of fixative agents (e.g. formalin) [37, 40, 42]. Bone specimens need further demineralisation prior to microtoming and slide preparation. These practical limitations are believed to contribute to the lack of histology co-location work with clinical bone sarcoma.

*Nonparallel* tissue dissection is more relevant to clinical workflows than parallel [31, 43], especially for sarcoma. Our solution uses the bisection identified on *ex-vivo* CT to introduce a reference surface, restoring the 3D spatial orientation of *nonparallel* 2D digitised histology slides, and facilitating further image volume registration.

Any histology co-location solution should collect data to support both soft tissue and bone segmentation. Emerging orthopaedic surgical technology aims to minimise damage to healthy soft tissue [2]. Confidence in image characterisation will be immediately useful in making resection decisions around nerves and blood vessels.

Previous work found that the presence of both bone and soft tissue in bone sarcoma specimens is problematic, but the rigidity of bone can be used to maintain tissue structure during cut-up without the need for external apparatus. Furthermore, the lower level of shrinkage of bone compared to other tissues [21] presents an opportunity to support robust co-location.

Clinical adoption of methods for prostate histology co-location has been slow for challenges in physical processing, lack of easy access methods for data combination, and time constraints in clinical pipelines [41]. Prostate cancer groups first shared their methodology and solutions for histology co-location [30, 31, 44] which informed subsequent clinical data collection [41, 43, 45]. Their solutions have continued to evolve and grow in recent years, where known histology slice correspondences have advised deep learning histology co-location [46].

Building on Phillips et al. sarcoma tissue processing methodology [1], we introduce a novel computational solution. Demonstrated on proof-of-concept *ex-vivo* animal data, it orients 2D histology within 3D radiology volumes and details the steps for histology image co-location in pre-surgical radiology.

**Fig. 2:**
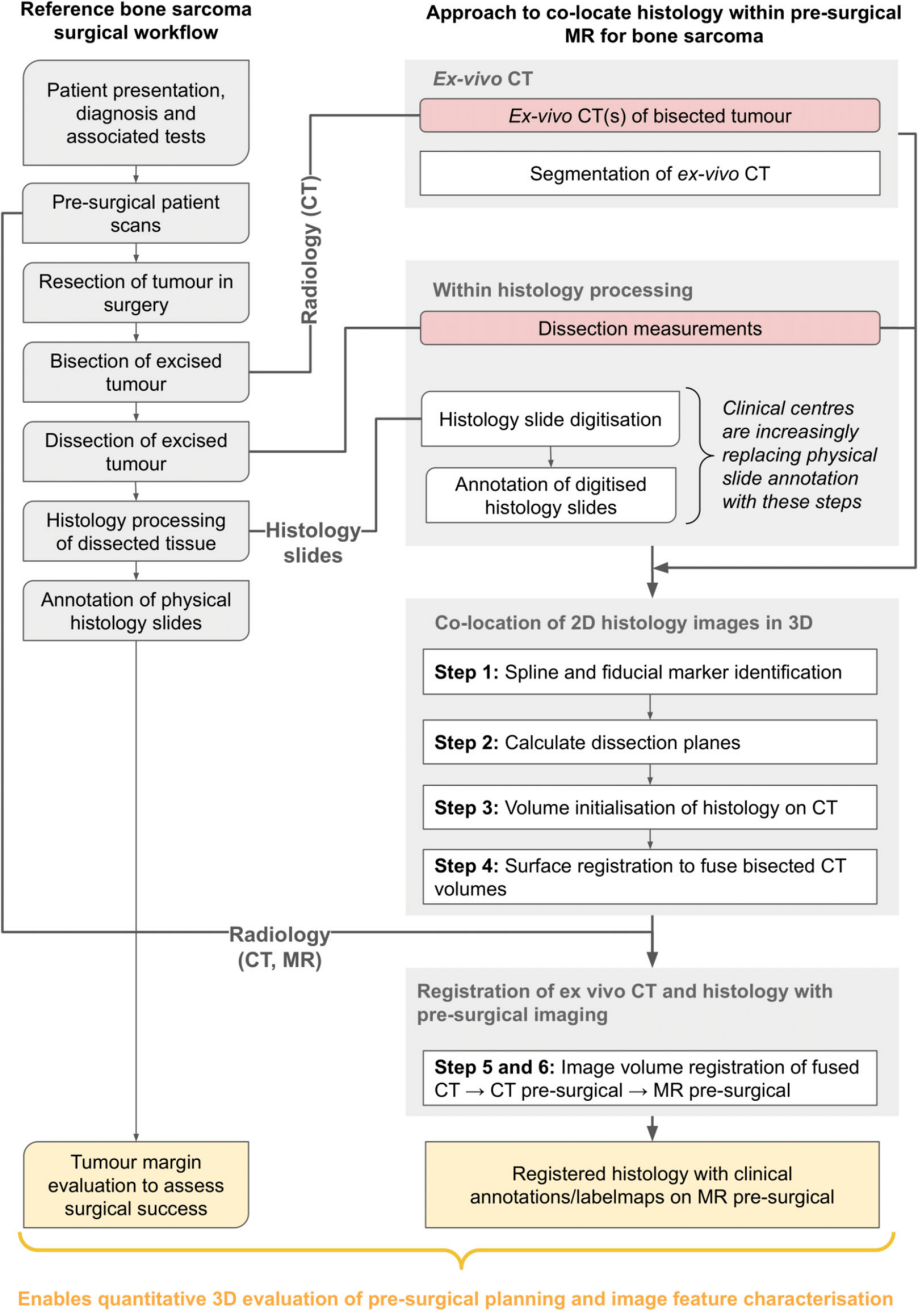
Image left is a reference sarcoma surgical workflow, while image right is an outline of the image co-location and registration steps this work presents. Lines between are where information is pulled from the surgical workflow. Red, rounded shapes are additional operations and data collection required in pathology processing

## Method

Extended consultation with surgeons, radiologists, and pathologists was the focus of previous work. This identified the requirements and constraints in their sarcoma workflows. See Fig. 2 for an overview of how the solution would fit into the reference surgical workflow.

### Steps in Solution Computations

#### Definitions

Note the term *‘bisection’* refers to approximately half a tumour specimen following the initial bandsaw cut but before *ex-vivo* CT. The term *‘dissection’* refers to tissue from subsequent bandsaw cuts of the two bisections after *ex-vivo* CT.

More definitions:

$CT_{post,1  \&  2}$: CT volume of each bisection
$H_{i}$: Digitised histology image for $i^{th}$ slide with associated label maps
$d_{a,i}$ [MYAMP: $d_{b,i}$ ] Laboratory measurements from fiducial reference ($f_{ref}$) to the two corners made by the bisection surface and each dissection cut
$P_{i}$: Assigned planes for $i^{th}$ dissection cuts (on which $H_i$ are initialised)
$H_{vol}$: Histology volume, featuring a sparse 3D surface of bone annotations
$T_{fuse}$: The transform to combine *ex-vivo* bisection CT volumes into $CT_{post, fused}$
$CT_{pre}$: Pre-surgical CT volume
$MR_{pre}$: Pre-surgical MR volume

#### Pre-processing

Bone segmentation in each $CT_{post}$ volume began with a threshold at 226 HU (Hounsfield Unit) [24] (default in some Materialise Mimics^©^ versions), followed by manual adjustment in 3DSlicer [47]. Segmentations and digital histology binary label maps were fed into a computational process outlined below. The annotated label maps were from QuPath [48].

**Step 1.**

**Fig. 3:**
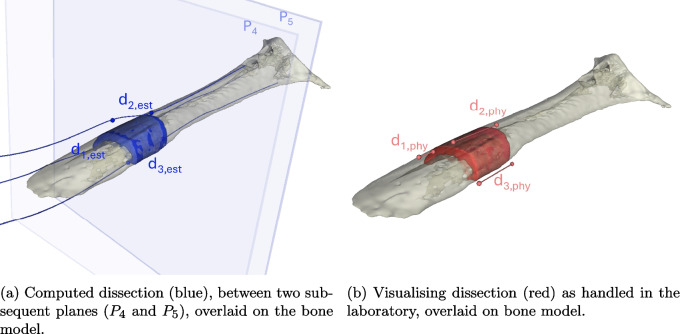
3D bone segmentation models of CT bisections. The Euclidean distances of spline intersection with subsequent splines ($d_{1,est}$, $d_{2,est}$, and $d_{3,est}$) were recorded along laboratory physical measurements ($d_{1,phy}$, $d_{2,phy}$, and $d_{3,phy}$) which were compared to evaluate plane assignment accuracy. Image generated using 3DSlicer

*Input:* Bisected bone segmentation for each $CT_{post}$ volume.

*Output:* The bisected bandsaw surface was contoured by two markup splines and a markup fiducial reference.

*Process:* Two 3DSlicer markups ($spline_{ma}$ and $spline_{mb}$) were manually placed along bone/fibrous tissue edges of the bisection as seen in the segmentation, with an additional markup point at $f_{ref}$. These were exported from 3DSlicer into separate files.

**Step 2.**

*Input:* The Cartesian position of $spline_{ma}$, $spline_{mb}$, and $f_{ref}$ control points.

*Output:* Planes assigned ($P_i$).

*Process:* Parameterised cubic polynomials were fit to $spline_{ma}$ and $spline_{mb}$ control points with coefficients stored. Each measurement $d_{a,i}$ and $d_{b,i}$ were treated as the Euclidean distance between $f_{ref}$ and the intersection of the $P_i$ with the cubic polynomials.

The Euclidean calculation was numerically solved thro-ugh fsolve of Scipy [49] using a variation on the Newton Raphson method to find the intersect in 3D of $P_i$ with the two polynomials.

At the resulting pair of intersections, parametric polynomials were differentiated. An average normal was calculated. This, combined with the vector between intersections, gave the cross product to define the geometry of dissection cuts as perpendicular to the curved bisection. This process modelled each approximate bandsaw dissection cut.

**Step 3.**

*Input:* $P_{i}$, $CT_{post}$ segmentations and $H_{i}$.

*Output:* Transforms to initialise $H_{i}$ into $H_{vol}$.

*Process:* This step involved out-of-plane rigid transformation of $H_{i}$ from the global import position to $P_{i}$ using the VTK GetObjectToWorldMatrix (3DSlicer terminal). Then 3 sets of landmark points were used to translate and scale binary histology label maps in-plane to overlay $H_{i}$ on each $CT_{post}$.

**Step 4.**

*Input:* Both $CT_{post}$ volumes and bone segmentations.

*Output:* $$CT_{post, fused}$$ volume.

*Process:* Each bisected cut surface as identified in **Step 1** segmentation was aligned using point-to-point fiducial registration (3DSlicer). Then multiple spherical surface points from the *dynamic modeller* module (3DSlicer) were used to refine the registration of the moving surface to the fixed model using *model registration* under the *IGT* module [50] (3DSlicer). This sequence of registrations combined to give $T_{fuse}$ for aligning bisected CT volumes to output a $CT_{post, fused}$ once run through the *stitch volumes* extension (3DSlicer).

**Step 5.**

*Input:* $CT_{post, fused}$ and $CT_{pre}$ volumes.

*Output:* Transform to register $CT_{post, fused}$, along with initialised $H_{vol}$, to $CT_{pre}$.

*Process:* The ‘generic (all)’ preset was used in Elastix [47] for combining canine volumes.

**Step 6.**

*Input:* $CT_{pre}$ and $MR_{pre}$ volumes.

*Output:* Transform to register $MR_{pre}$ to $CT_{pre}$. Alternatively, the total transform could be inverted to register $CT_{pre}$ and associated $H_{vol}$ to $MR_{pre}$.

*Process:* The Elastix [47] module was again used with the same preset for this.

### Testing Plane Assignment (of Step 1 and 2)

It is important to appreciate misalignment in histology co-location [51]. We tested this by comparing laboratory and computed measurements of dissected tissue size for three amputated canine limbs (one tumorous and two non-tumorous canine limbs, with two and four bisections, respectively). Figure 3 illustrates how dissection size measurements were collected as proxies for the relative positions of the dissection cuts to understand translation and rotation errors in plane assignments.

We paired Euclidean distances between original spline intersections on successive planes ($d_{1,est}$ and $d_{3,est}$) with physical Vernier calliper measurements ($d_{1,phy}$ and $d_{3,phy}$) from the laboratory. Similarly, we compared Euclidean distances along a third spline ($d_{2,est}$) that contoured the surface of bone and fibrous tissue on *CT* with a third set of calliper measurements (${d_2,phy}$) for each dissection.

The sensitivity of plane assignment to manual spline input was further evaluated. Expert interpretation determined the extreme placement of the splines on bone or fibrous tissue in $CT_{post, 1 \& 2}$. The planes $P_{i}$ calculated from these extreme spline inputs were compared to understand how this manual operation affected translation and rotation.

### Proof-of-Concept

#### Data Description

The clinical data used in this work was of amputated canine limbs. Owner consent was obtained for the use of the data in education and research prior to collection. Routine veterinary treatment remained unchanged, as all imaging and processing were performed after amputation. This work was granted an exception for animal ethics approval because no investigations were performed on, and no treatment was altered for live dogs.

All images, animal and owner details, were de-identified with DicomCleaner (PixelMed Publishing$^{TM}$) for solution development and testing. Imaging of amputated limbs served as an acceptable surrogate for pre-surgical imaging due to minimal anatomical and morphological changes in this type of tumour. Clinical canine specimens were considered a suitable model for humans because canine bone tumours have many clinical similarities to equivalent human disease [52].

##### Experimental Radiology Protocols

Multi-parametric MR and CT data was available from previous work to assist solution development. Additional image stacks from a 3T Magnetom Vida (Siemens Healthineers, Germany), Christchurch City, Ōtautahi, New Zealand were used to evaluate the solution in the proof-of-concept. This work used 1 mm slice sagittal spatial reconstructions highlighting bone (T1) and tumour (T2 Turbo Spin Echo). MR sequence parameters were adjusted for scanning a small FOV.

High-definition CT of the whole limb ($CT_{pre}$) and bisected specimens ($CT_{post,1}$ & $CT_{post,2}$) were taken on a Revolution GSI (GE Medical Systems, USA), Lincoln, New Zealand. This work used 0.625 mm slice axial spatial reconstructions. These were acquired at 120 kVp with a 0.5 pitch and 512 $\times$ 512 matrix.

##### Experimental Histology Digitisation

Standard-size Haematoxylin and Eosin (H&E) stained histology slides were digitised by Awanui Labs in Christchurch City. Large format H&E slides were digitised at 12.5 X zoom with a scanning optical camera setup as no cassettes were present in the country for digitising large-format histology on any commercial scanner.

A human clinical pathologist annotated all digital histology images while referencing physical slides under a microscope. However, as high-resolution commercial digitisation systems become more available [53], referencing of physical slides may become less relevant.

## Results

**Fig. 4:**
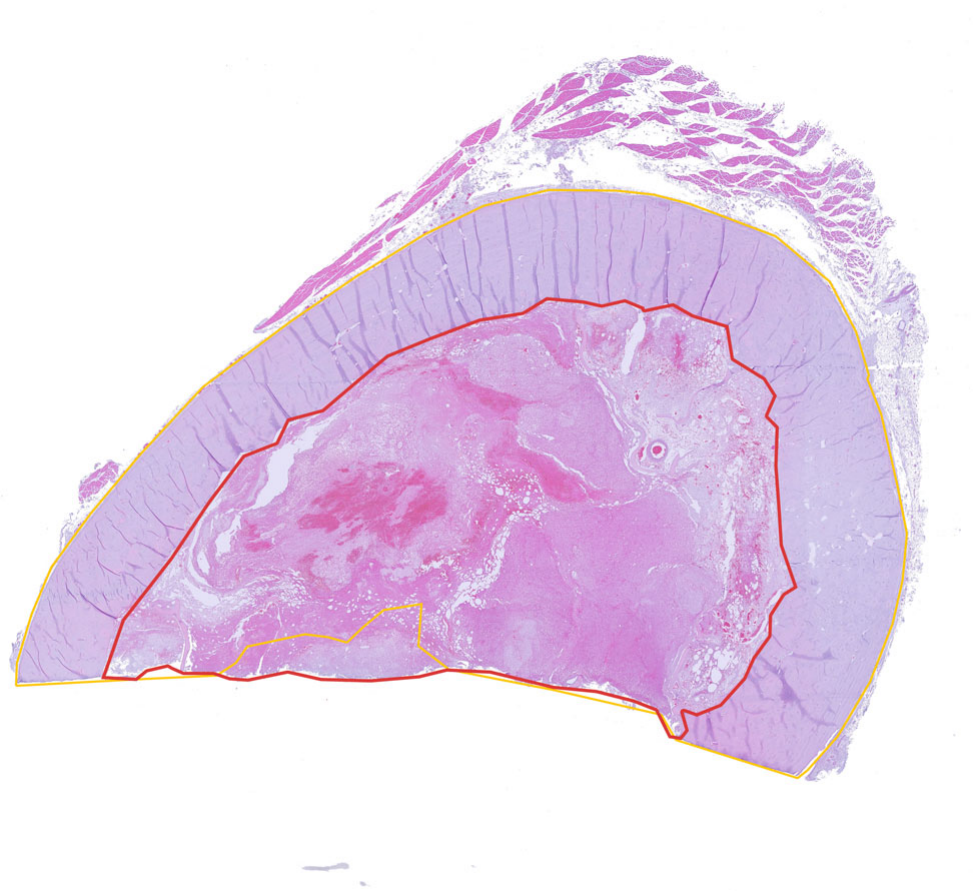
Image of histology slide with (yellow) bone and (red) tumour label maps. Image and label maps generated from QuPath

### Testing Plane Assignment (of Step 1 and 2)

Our study evaluated the accuracy of plane assignment by comparing computed measurements and physically dissected tissue size. Across 114 measurements on 38 dissections (over 6 bisections), the mean error difference was 0.19 mm (± 1.8 mm). Additionally, the rotational analysis showed a mean error difference of 0.25 mm (± 1.9 mm) for $d_2$ measurements and 0.4 mm (± 1.6 mm) for $d_1$ and $d_3$ measurements, based on 38 and 76 measurements, respectively.

Comparison of plane assignments from different extreme user spline inputs showed a maximum rotational sensitivity of 1.6°and a mean translation sensitivity of 0.08 ± 0.2 mm (*n* = 114), based on the variation in plane-to-plane distance of spline intersections.

**Fig. 5:**
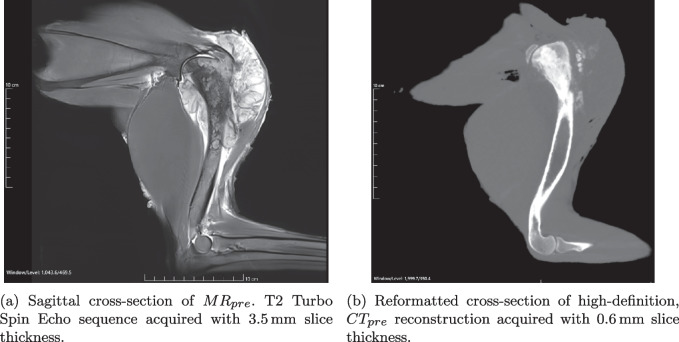
Radiology of whole proof-of-concept canine forelimb taken on clinical equipment with pre-surgical imaging protocols. Images generated using Weasis

**Fig. 6:**
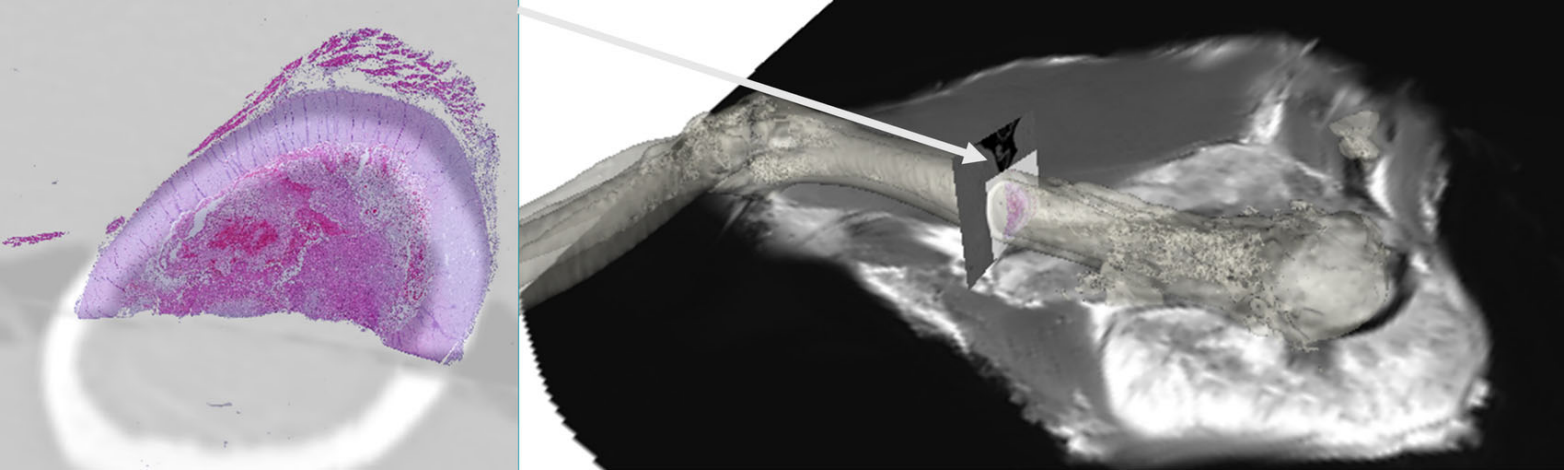
Image left is a single digitised axial histology image overlaid on axial $CT_{post, fused}$ cross-section. Image right is the same CT and overlaid histology orientated and overlaid on the bone segmentation of $CT_{pre}$ (opaque), along with a sagittal cross-section of $MR_{pre}$. Image generated by 3DSlicer

### Step 3 and Beyond

Histology images and annotations Fig. 4 have been co-located in two *ex-vivo* CT volumes and subsequently registered to $CT_{pre}$ and $MR_{pre}$ Fig. 5. In the proof-of-concept tumour specimen, histology images were roughly orthogonal to the humerus bone as shown in Fig. 6.

Note that histology image orientation is restricted to $P_{i}$. The orientation of which depends on tissue processing decisions and measurement in pathology.

## Discussion

We present a solution that enables the co-location and registration of 2D sarcoma histology images with pre-surgical CT and MR 3D volumes. This solution utilises *ex-vivo* CT of resected tissue and basic laboratory measurements to restore the 3D orientation of tissue in histology, which is largely lost during current pathology processing.

In **Step 1**, the solution maintains dependence on user-guided CT bone segmentation as described in the “Pre-processing” section. Fortunately, prior research has demonstrated high accuracy in CT bone segmentation, overestimating the bone boundary with a Root Mean Square (RMS) error of 0.55mm [24].

Direct measurement of rotational or transnational plane assignment error was not feasible in the present study. Error was assessed for **Steps 1** and **2** by comparing measured dissection size against the Euclidean distance between spline intersections with consecutive planes. There was troubling uncertainty in the measurement discrepancies because they did not initially follow a normal distribution.

This uncertainty seems to have originated, at least partially, from the assumption of flat dissection cuts, whereas pathologists recorded some as curved. The exclusion of curved dissection data supported normality (Shapiro-Wilk score of 0.367), supporting confidence in the current plane assignment validation method.

Errors between $d_2$ measurements showed higher variance than the measurements on bisection edges ($d_1$ and $d_3$), indicating rotational error in plane assignment. The perpendicular dissection assumption is believed to be weak and worthy of further testing, especially for heavily concave bisections.

The wide resection margins in current clinical use require surgical cuts to remove at least 10 mm of healthy tissue [6, 54]. Acceptable margins in histology can be down to 2 mm macroscopically [22, 54] and even smaller microscopically. With 0.19 mm translation and 0.25 mm rotational error from plane assignment, interpretation of anatomical measurements and tumour margins in co-located histology is expected to remain smaller than current wide bone sarcoma resection margins. Thus, co-located histology is expected to support enhanced radiologic interpretation, allow better prediction of histological outcomes during pre-surgical planning, and support the use of smaller resection margins with evolving surgical technology (e.g. PSI). However, further clinical validation of this proof-of-concept work is required.

Plane assignment errors are comparable to target registration errors (TREs) in prostate research (0.71 mm) [31]. However, the use of different evaluation methodologies means that this comparison should be interpreted with caution. More specimens are needed to perform TRE evaluations using anatomical landmarks on histology ima-ges.

Future work may choose to evaluate histological processing and tissue deformation assumptions similar to how [31, 51] quantified the TRE and found co-location misalignment to be 1.1–1.9°and 0.9–1.3 mm [51] by using anatomic landmarks on histology images. Ongoing image processing work aims to explore the use of bone label maps in histology initialisation (**Step 3**) to implement out-of-plane histology deformation and image co-registration quantification.

The authors would like to note that the physical deformation of bone tissue during histological processing is less than that of other tissue [21]. This potentially offers a degree of stability in physical histology processing above tissue types used in co-location work to date.

According to current clinical guidelines, initial tumour dissection should occur in the plane of the largest dimension, typically longitudinal, followed by axial sectioning [40]. The proof-of-concept specimen processed for this work did not undergo longitudinal dissection processing before axial cuts were made. However, the solution is flexible and can be adapted to meet specific guidelines. Where a longitudinal dissection could provide data for a digital histology mosaic to be registered parallel to the bisection before axial histology images are registered at an offset, orthogonal to the bisection surface.

For this work, the thickness of microtome shavings was used as a plane offset in geometric calculations. Additionally, the width of the bandsaw blade can be used to assign planes if measurements are taken to the blockface after dissection. Decisions on how to include these measurements will need to consider the orientation of the bisection during cut-up.

Laboratory guidelines have a necessary level of ambiguity for processing more complex bone sarcoma specimen geometries. The ultimate responsibility of cut-up falls to pathologists [40], not guidelines. Therefore maintaining a clear dialogue between staff registering digital histology, pathologists and those performing cut-up (if different personnel) is crucial to ensuring collected measurements are of value.

Laboratory technicians communicated it was relatively easy to measure dissection cuts from the tactile corner of a fibrous tumour. However, it was challenging to delineate fibrous tissue from other soft tissue on CT for spline placement. Across the extreme proof-of-concept tumour case (approximate tumour mass spanned 16 cm from proximal to distal) and the two non-tumorous specimens, extreme input markup splines resulted in 0.08 ± 0.2 mm of relative translational sensitivity and 0°to 1.6°rotational plane sensitivity of $P_{i}$. This analysis shows the solution’s plane calculation sensitivity to manual spline assignment, but more specimens and data are needed for a thorough evaluation.

For this work, it was assumed that a usable fiducial reference point could be created or was already present. A drill hole was used in processing proof-of-concept tissue however future work could use a joint condyle or the corner of bone formed by resection cuts (e.g. in chevron osteotomy). These alternatives have been shown to be identifiable on radiology and histopathology in related sarcoma work [26].

Proof-of-concept data has been collected to demonstrate the continuity of the solution. Some tissue needed reprocessing due to MR imaging equipment constraints and fixation issues, causing delays, excessive shrinkage, and lower quality in some histology images. Although not suitable for characterising radiographic features, the data is available on request to further understand the work and replicate the solution.

The rarity and heterogeneity of bone tumours posed a challenge, similar to previous research [17, 26]. We used one tumorous canine specimen to develop our proof-of-concept solution, then one additional tumorous and two non-tumorous specimens for demonstration and testing. The use of these three specimens has enabled the plane assignment testing and demonstrated the solution. However, more specimens and data are needed for more rigorous future validation.

Using animal tissue enabled efficient prototyping and initial testing, but the need for dedicated whole limb CT and MR with animal specimens introduced logistical challenges. Since $CT_{pre}$ and $MR_{pre}$ are already routine in human bone sarcoma treatment workflows, we have assumed that it should be available in future research.

In this work, a custom *stitch volumes* extension parameter set was made from the 5.6.2 3DSlicer release for processing the proof-of-concept CT bisections. This adjusted parameter set includes three modified lines and can be found in the StitchVolume file in the linked repository.

Alternatively, if customising any parameter set is not possible, future 3DSlicer releases will offer a new *StitchVolumes* extension. This extension includes a ‘Stitch Weighting Method’, which can perform **Step 4** equivalently with a 0 Voxel threshold. The effort needs to be directed towards quantifying the volumetric registration of histology to *ex-vivo* CT, and ultimately *in-vivo* MR. Histology image deformation and manipulation are ongoing works but complete quantification of histology co-location will require more data.

No significant effort was put into developing a particular extension or software package for rigid, deformable or other volume-to-volume image registration because other research groups are making rapid advances in this area [55]. It is noted that pre-surgical CT and MR registration is particularly useful in complex musculoskeletal tumour resection planning [23, 56]. However, advice from regional professional clinical organisations should be used to guide the registration software used in sarcoma treatment centres [55, 57]. Ultimate histology co-location quantification will need to consider the error incurred by volume-to-volume registration.

### Development Opportunities

Some studies have reported that deep learning is a superior tool for histology co-location in certain tissues [46]. However, most existing initiatives focus on histology that has been sliced in *parallel* and assumes slice correspondence. When this is not clinically feasible, as in the case of bone sarcoma, additional orientation information is required. The use of *ex-vivo* CT and laboratory measurements can inform *nonparallel* co-location of bone sarcoma histology as demonstrated through our solution. Future co-location work with sarcoma histology may consider deep learning as a tool, similar to how existing research [46] has used reference methods [41, 44]. We freely share a solution as a reference method base for future research to advance clinical human and veterinary sarcoma histology co-location.

While digitisation of large-format (or whole-mount) histology is rare, mosaicing techniques only require standard-size histology images. A variety of software options for this purpose are listed by [29]. With our solution, we stress that proper labelling of dissection orientation is essential, particularly if standard-sized histology is to be processed. Surgical dye, often used in many surgical workflows, has proven useful in maintaining specimen orientation during proof-of-concept bisection and dissection.

The assignment of $P_i$ on $CT_{post,1 \& 2}$ volumes and the use of clinical histology labels as collected for the proof-of-concept specimen will assist histology deformation. Deformations might employ techniques such as free-form deformations (e.g. RAPSODI) [41]. The authors do note that non-mineralised osteoid could not be easily demarcated from mineralised osteoid in proof-of-concept histology [58] due to necessary demineralisation in pathology processing. This is expected will introduce error if deformations assume all osteoid in bone histology label maps is radiodense.

Single energy CT’s reliance on density for tissue identification restricts it to including only mineralised osteoid in bone segmentation. Multi-energy CT may prove promising at differentiation between areas of non-mineralised osteoid and extraneous soft tissue. However, research focusing on this issue has not been found. An intermediary quantification of deformation error with any radiodense osteoid assumption would be worthy of future investigation.

### Opportunities for Wider Healthcare and Technology

Co-location of 2D clinical histology images within 3D radiology volumes will give physicians and researchers the opportunity to contribute cohesive datasets of spatially characterised, microscopic tissue label maps to support radiology interpretation and surgical technology development. It will make high-fidelity tissue label maps of a clinical standard available for training segmentation algorithms at minimal additional cost. Integrating *ex-vivo* bisection CT and Euclidean measurements while processing proof-of-concept tissue has been shown can do this.

Datasets of co-located histology and radiology will enhance the accuracy of volumetric segmentation by making histology insights available earlier in the bone sarcoma presentation-diagnosis-planning-surgery-recovery sequence. This will support margin decisions for surgical resection of bone sarcoma with evolving, patient-specific technologies.

## Conclusion

In this research, we present a solution to co-locate clinical bone sarcoma histology images in pre-surgical CT and MR radiology volumes. The solution is specifically engineered to convey resilience to the complexities of sarcoma histology processing. It uses *ex-vivo* CT of removed tumour tissue and laboratory measurements. A proof-of-concept bone sarcoma tumour has been processed to affirm end-to-end suitability in the clinical context.

The computations described with this solution are capable of spatial, *nonparallel* histology image co-location with an accuracy of 0.19 ± 1.8 mm and user sensitivity of 0.08 ± 0.2 mm. Continued collection of validation measurements ($d_{phy}$) in pathology will allow further evaluation of inherent inaccuracies from user input and histology image orientation assumptions.

This work complements other histology co-location work, with the possibility to build upon it [41]. It leverages existing tools in 3DSlicer and contributes a robust solution to the literature for co-location of histology images, and ultimate registration within 3D radiology.

A routine combination of tumour histology and radiology through this solution will facilitate the assessment of existing treatment plan accuracy and pave the way for optimising radiology interpretation schemes. It will support the continued evaluation of safe pre-surgical resection margins in bone sarcoma treatment.

## Data Availability

Proof-of-concept data is available from the corresponding author upon reasonable request.

## Code Availibility

Python code for the project has been publicly shared at https://github.com/phiro753/Py-OrthoRadPathFusion.
